# Case Report: Loss-of-Function *ABCC9* Genetic Variant Associated With Ventricular Fibrillation

**DOI:** 10.3389/fgene.2022.718853

**Published:** 2022-04-13

**Authors:** Anastasia Zaytseva, Tatyana Tulintseva, Yulya Fomicheva, Valeria Mikhailova, Tatiana Treshkur, Anna Kostareva

**Affiliations:** ^1^ Almazov National Medical Research Centre, St Petersburg, Russia; ^2^ Sechenov Institute of Evolutionary Physiology and Biochemistry, Russian Academy of Sciences, St Petersburg, Russia; ^3^ Department of Woman and Child Health, Karolinska Institute, Stockholm, Sweden

**Keywords:** *ABCC9*, atrial hypertension, case report, K_ATP_ channels, ventricular fibrillation (VF)

## Abstract

Genetic variants in the *ABCC9* gene, encoding the SUR2 auxiliary subunit from K_ATP_ channels, were previously linked with various inherited diseases. This wide range of congenital disorders includes multisystem and cardiovascular pathologies. The gain-of-function mutations result in Cantu syndrome, acromegaloid facial appearance, hypertrichosis, and acromegaloid facial features. The loss-of-function mutations in the *ABCC9* gene were associated with the Brugada syndrome, early repolarization syndrome, and dilated cardiomyopathy. Here, we reported a patient with a loss-of-function variant in the *ABCC9* gene, identified by target high-throughput sequencing. The female proband presented with several episodes of ventricular fibrillation and hypokalemia upon emotional stress. This case sheds light on the consequences of K_ATP_ channel dysfunction in the cardiovascular system and underlines the complexity of the clinical presentation of *ABCC9*-related diseases.

## Introduction

The *ABCC9* gene encodes a transmembrane protein SUR2 that forms the regulatory part of the ATP-sensitive potassium channel (K_ATP_) in cardiac, skeletal, vascular, and nonvascular smooth muscle cells (Chutkow et al., 1996). K_ATP_ channels are heterooctamers, which consist of four pore-forming K_ir_6.x channels, associated with four regulatory SUR (sulfonylurea receptor) subunits. Their main function is to couple the cell metabolic state to its membrane potential, thus adapting the K^+^ conductance to the ATP content *via* the ATP-specific channel blockade [for review see (Foster and Coetzee, 2016)]. The subunit composition of K_ATP_ channels depends on the tissue subtype. There are two main types of SUR splice variants (SUR2A and SUR2B), which contribute to the diversity of K_ATP_ channels (Chutkow et al., 1999). There are two main types of K_ir_6.x channels—K_ir_6.1 and K_ir_6.2—encoded by *KCNJ8* and *KCNJ11* and two SUR proteins—SUR1 and SUR2—encoded by *ABCC8* and *ABCC9* genes, respectively (Aguilar-Bryan et al., 1995; Inagaki et al., 1995; Chutkow et al., 1996).

The gain-of-function (GOF) and loss-of-function (LOF) mutations of all genes encoding K_ATP_ subunits (*ABCC8, KCNJ11*, *ABCC9,* and *KCNJ8*) have been described in patients with completely different and surprisingly opposite phenotypes. The regulatory subunit SUR1 encoded by *ABCC8* and the pore-forming subunit K_ir_6.2 encoded by *KCNJ11* are mainly co-expressed in insulin-secreting tissues. Their GOF mutations are associated with neonatal diabetes, while the LOF mutations cause hyperinsulinism (Galcheva et al., 2019; Pipatpolkai et al., 2020). No cardiovascular phenotype has been reported for SUR1-and *ABCC8*-associated diseases. The regulatory subunit SUR2 encoded by *ABCC9* and pore-forming subunit K_ir_6.1 encoded by *KCNJ8* are expressed in cardiomyocytes, vascular smooth muscles, endothelial cells, and in many other cells and tissues with different presentations of SUR2A and SUR2B isoforms. *ABCC9* and rarely *KCNJ8* GOF genetic variants are associated with the Cantu syndrome, which often presents with the cardiac phenotype (Grange et al., 2019). Although the most typical and recognized clinical signs of Cantu syndrome include hypertrichosis and a characteristic facial appearance with acromegaloid features, the cardiovascular system’s involvement often occurs in the form of cardiomegaly with normal cardiac function, patent ductus arteriosus (PDA), and dilated aortic root (Levin et al., 2016). Additionally, several GOF genetic variants in the *KCNJ8* gene were reported in association with the isolated Brugada syndrome, atrial and ventricular fibrillation, and early repolarization syndrome (Haïssaguerre et al., 2009; Medeiros-Domingo et al., 2010; Barajas-Martínez et al., 2012; Delaney et al., 2012). In contrast, there have been several cases of LOF genetic variants in *ABCC9* and *KCNJ8*. Tester and co-authors described and functionally characterized *KCNJ8* LOF variants in a cohort of sudden infant death syndrome victims in 2011 (Tester et al., 2011). *ABCC9* LOF variants have been reported only twice in a patient with dilated cardiomyopathy and in a case of isolated atrial fibrillation (Bienengraeber et al., 2004; Olson et al., 2007). There were no additional reports on *ABCC9* LOF variants within the next 13 years. The clinical significance and accurate genotype–phenotype correlations of LOF variants in this gene remain unclear. Recently, a homozygous LOF genetic variant in the *ABCC9* gene has been described as a cause of a novel phenotype called intellectual disability and myopathy syndrome (Smeland et al., 2019).

Here, we report another case of a LOF *ABCC9* genetic variant associated with idiopathic ventricular tachycardia and arterial hypertension. The presented case further extends the phenotypic spectrum of *ABCC9*-related disorders and supports the role of K_ATP_ channels in cardiomyocyte and coronary smooth muscle electrophysiology and function.

## Clinical Report

The study was conducted according to the guidelines of the Declaration of Helsinki and approved by the Institutional Ethics Committee of the Almazov National Medical Research Centre. Informed consent was obtained from all subjects involved in the study. Routine clinical examination was performed according to the standard protocols including electrocardiography, Holter monitoring, echocardiography, endomyocardial biopsy, and biochemical and hormonal tests. For genetic testing, a targeted panel of 172 cardiomyopathy-associated genes was analyzed using the SureSelect Target Enrichment System and Illumina MiSeq instrument (Agilent; Waldbronn, Germany). The list of studied genes is presented in Supplementary Table S1. The data processing and filter strategy were performed as described earlier (Jorholt et al., 2020).

**FIGURE 1:**
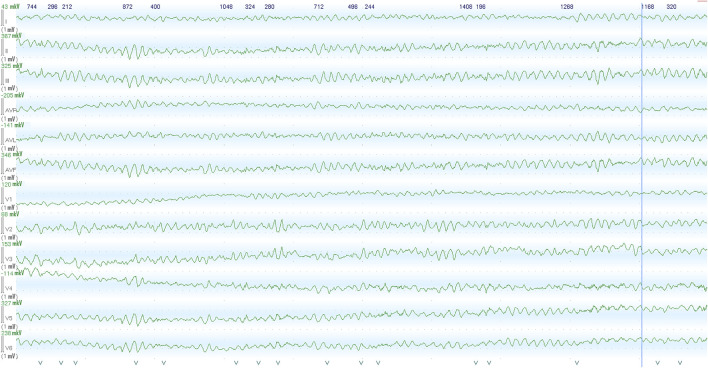
Images recorded by the ECG monitoring system in the intensive care unit within the first hour after the initial out-of-hospital episode of abortive sudden cardiac death. Ventricular fibrillation was recorded from patients, following admission to cardiac ICU.

**FIGURE 2:**
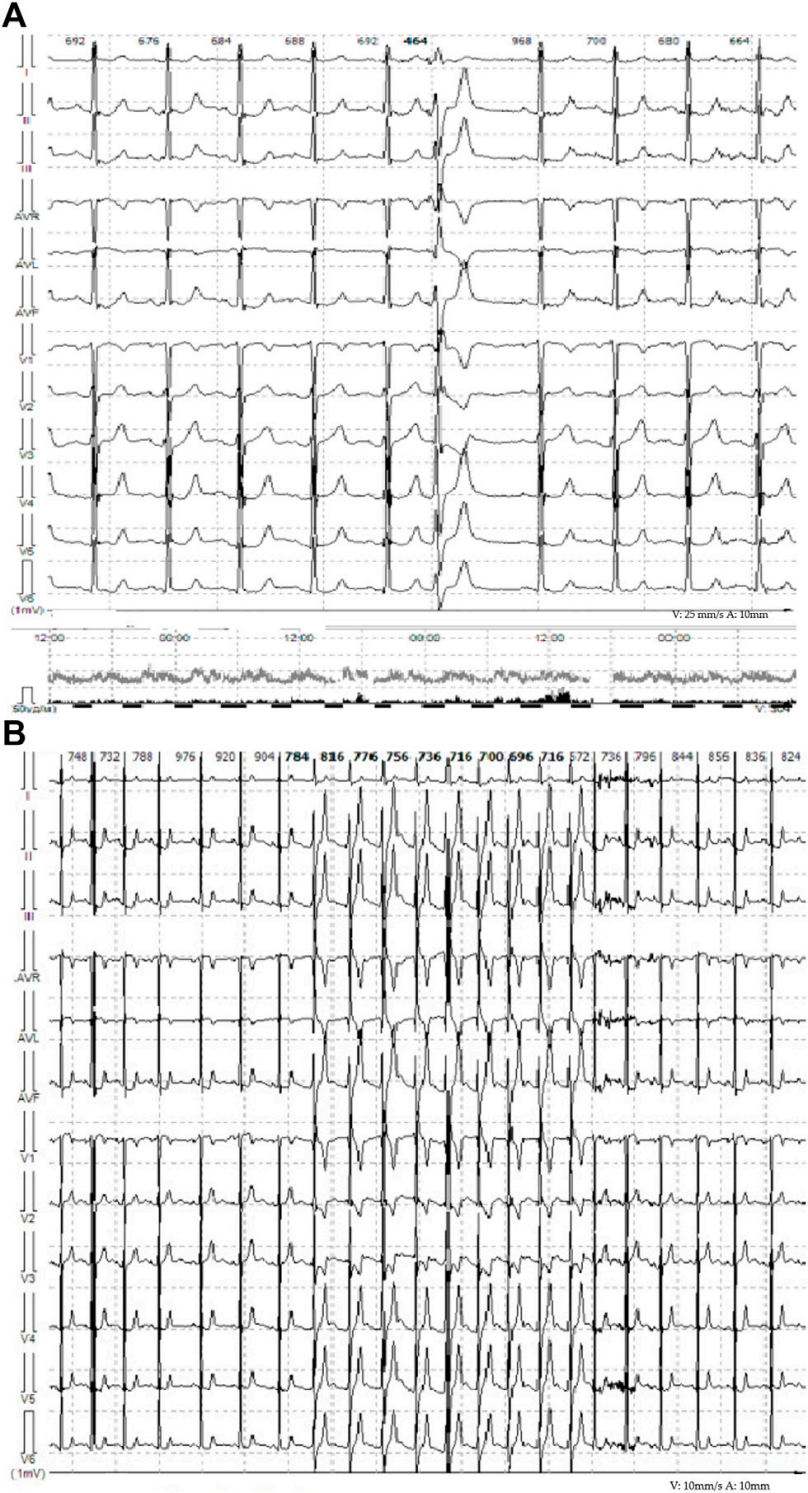
**(A)** Representative ECG from telemonitoring, single monomorphic ventricular ectopic complexes. **(B)** Representative ECG from telemonitoring. Unstable episodes of the monomorphic idioventricular rhythm with frequency of 81 bpm are observed.

The proband – 32-year-old female patient with a previously uneventful life history presented with a sudden cardiac death that occurred outside the home after emotional stress. The patient was resuscitated by her husband until the emergency services arrived when ventricular fibrillation (VF) was registered (Figure 1). Effective defibrillation led to the recovery of sinus rhythms, but within the next 30 min, in the intensive care unit, several episodes of VF repeatedly occurred registered on the ECG monitoring system, which lead to the deep sopor and clonic seizures. The patient’s blood tests revealed hypokalemia (2.9 mmol/L) with additional remarkable changes. There were no alterations in the hormonal state, glucose metabolism, the concentration of electrolytes, and no evidence of endocrine genesis of arrhythmia. Acute cerebrovascular accident and myocardial injury were excluded. On the resting ECG on the first day after VF, changes in repolarization (-) T in I, aVL, and V1-V3 leads were recorded, not subsequently detected. During 15 days of ECG telemonitoring, the sinus rhythm was recorded with an average heart rate of 68 bpm. Single monomorphic ventricular ectopic complexes (*n* = 3,185) were found mainly in the daytime, and 11 unstable episodes of the monomorphic idioventricular rhythm with an average frequency of 92 bpm were registered for the entire period of telemonitoring (Figures 2A,B). There were no changes in the QT interval. Echocardiography demonstrated the slightly increased LV wall thickness (13 mm) and increased myocardium mass (92 g/m2) corresponding to concentric myocardial hypertrophy with a normal ejection fraction (65%). Cardiac MRI revealed no signs of myocarditis or arrhythmogenic dysplasia, normal chamber geometry, normal ejection fraction (62%), and an absence of a late gadolinium enhancement phenomenon.

**FIGURE 3:**
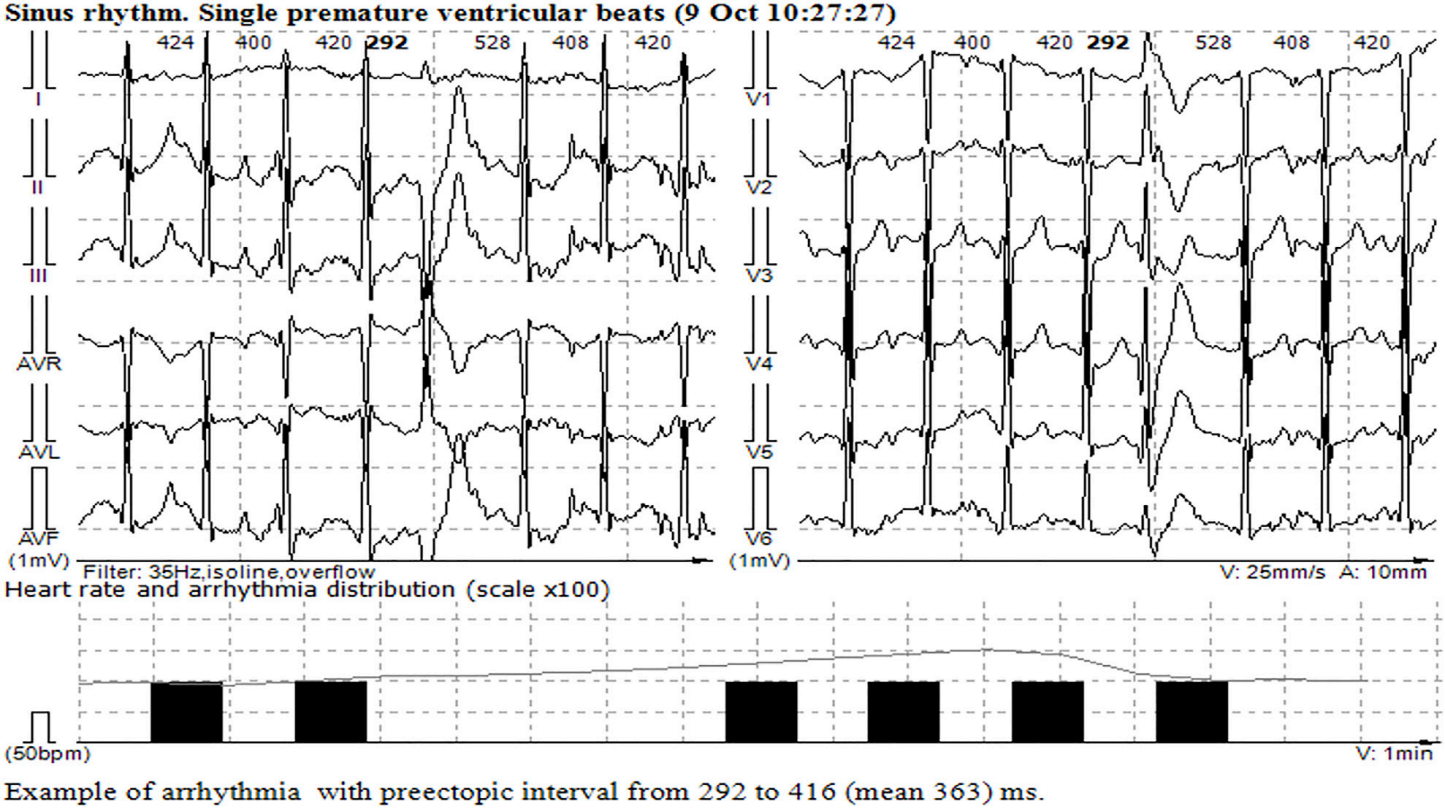
Single monomorphic ventricular ectopic complexes during and at the peak of stress echocardiography.

The patient remained on ventilation support during the next 5 days, followed by successful restoration of spontaneous respiration and consciousness. ECG normalized on the seventh day after hospitalization; the number of single ectopic ventricular complexes reduced to 500 per day, and ventricular tachycardia and QT interval prolongation were not detected. The discharge with bisoprolol 5 mg/day was recommended. Stress echocardiography performed several months later demonstrated high exercise tolerance and no myocardial ischemia, but at the peak of physical activity, single ectopic ventricular complexes were registered (Figure 3). The implantation of the implantable cardioverter defibrillator was performed for secondary prevention, and the patient remained stable within the next 2 years on metoprolol therapy (100 mg daily).

According to the anamnesis, the patient developed normally and has an average intelligence. The patient did not have any difficulties in learning at school and demonstrated standard communication skills. The patient had no history of chronic diseases and no family history of sudden cardiac death. Holter monitoring performed during the pregnancy 6 years prior to the episode of abortive sudden death documented 2,683 single ventricular ectopic beats. The patient had three natural self-deliveries with preeclampsia (moderate degree) and arterial hypertension (up to 160/100 mmHg) during the third pregnancy, and arterial hypertension retained after the delivery. No causes of the secondary hypertension were found; the patient remained normotensive on ACE inhibitor treatment with rare hypertensive crises upon intensive physical excises or emotional stress.

To exclude the inherited arrhythmic syndromes or early manifestation of cardiomyopathy, the genetic study was performed using a targeted gene panel of 172 cardiomyopathy-associated genes, as previously described (Jorholt et al., 2020). All disease-related genetic variants were confirmed using Sanger sequencing and classified according to the American College of Medical Genetics guidelines (Jorholt et al., 2020). Informed consent was signed prior to the investigation, and the study protocol was approved by the Ethical Committee of the Almazov National Medical Research Centre. All research studies have been performed in accordance with the Declaration of Helsinki.

The target panel sequencing identified a genetic variant in the *ABCC9* gene in the exon 37 *ABCC9* (NM_005,691.3):c.4570_4572delTTAinsAAAT (p.Leu1524LysfsTer5), rs869025349, classified as pathogenic and earlier reported in a family with dilated cardiomyopathy and ventricular arrhythmias. Previous characterization of recombinant K_ATP_ channels including this mutation suggests that the variant causes a decrease in current expression, suggestive of a LOF molecular phenotype (Bienengraeber et al., 2004). This variant was confirmed in proband’s father, sister, and young daughter but not in the mother and older daughter (https://varsome.com/variant/hg19/chr12-21958186-TAA-ATTT). The genotype-positive young daughter experienced several syncope episodes at age 12, but no ECG was registered at that time, and the precise link of these episodes to the arrhythmogenic events remains unclear. This variant was previously reported in ClinVar in connection to dilated and arrhythmogenic cardiomyopathy but is currently classified as a variant of unknown significance. However, the absence of this variant in the gnomAd exome or gnomAd genome database together with the loss of function nature and several reported clinical associations prompts to reclassify the variant as pathogenic according to ACMG guidelines. The only reported frequency available for this variant comes from the Kaviar database, where it was reported with relatively high frequency among 454 genomes from the Wellderly study (00,032%), but these data have not been confirmed in other datasets. Therefore taking into account gnomAd exome and genome data, the true frequency of rs869025349 seems to be very low. No other genetic variants, characterized as pathogenic or likely pathogenic, was identified in the proband.

In spite of the fact that the described genetic variant potentially has a LOF mechanism of action different from that in GOF variants linked to the Cantu syndrome, we prompted the deeper clinical phenotyping in order to search for Cantu-related clinical features and excluded the possibility of a mixed clinical phenotype linked to *ABCC9* variants. The patient revealed no signs of hypertrichosis, dysmorphic face, or enlarged acromegaloid features. The patient did not present with reduced motor skills or delayed development in childhood. Unlike the characteristic features observed in the autosomal recessive SUR2 LOF syndrome, AIMS, no hypotonia, skeletal abnormalities, or scoliosis were noted neither in the proband nor in other carriers of the L1524KfsTer5 variant. The patient did not complain of muscle pain or fatigue after physical exercise. All carriers of the aforementioned genetic variant had an average intelligence and no difficulties in school learning; the patient did not reveal any skin lesions or pathologies and did not complain of sleep apnea. However, no specialized somnological examination has been performed.

## Discussion

K_ATP_ channels are of key importance in metabolic stress sensing of the cell. The expression of these channels has been found in various organs and tissues, including the pancreas, nervous system, skeletal and smooth muscle cells, and cardiac myocytes (Miki and Seino, 2005). These channels are composed of pore-forming and regulatory subunits; the latter in the heart is represented by SUR2 protein encoded by the *ABCC9* gene. This gene is a member of the superfamily of the adenosine triphosphate (ATP)-binding cassette (ABC) transporter subfamily C, member 9, which is located on chromosome 12 at 12p12.1^1^. The main isoform of SURs in the cardiac and vascular myocytes, SUR2A, consists of 17 transmembrane segments, organized in three domains: TMD0, TMD1, and TMD2. In addition, there is a highly conserved intracellular region called nucleotide-binding domain 1 (NBD1) with Walker A and Walker B motifs in the linker between TMD1 and TMD2. Another nucleotide-binding site of SUR2A is the NBD2 region localized in the C-terminal part. It is suggested that NBDs are responsible for channel activation (Foster and Coetzee, 2016).

K_ATP_ channel dysfunction leads to the abnormal cellular response to metabolic stress. The growing amount of evidence indicates that K_ATP_ plays an important role in the adaptive cardiac response to systemic metabolic stressors and vascular tone regulation. Numerous cardiac K_ATP_ GOF mutations have been described in the association with Cantu syndrome (Levin et al., 2015) giving a broad range of molecular pathophysiological events linked to K_ATP_ GOF (Huang et al., 2018; McClenaghan et al., 2018). In contrast, LOF mutations in the *ABCC9* gene were only a few times reported in association with quite distinct clinical phenotypes due to different allelic states (heterozygous mutations (Bienengraeber et al., 2004; Olson et al., 2007; Hu et al., 2014) and homozygous mutation reported by Smeland et al. (2019). Therefore, the frequency and clinical consequences of *ABCC9* LOF genetic variants have not been thoroughly characterized until now. Here, we reported a patient with a LOF variant in the *ABCC9* gene (Leu1524LysfsTer5, rs869025349) associated with a complex clinical phenotype, including arterial hypertension and stress-induced ventricular arrhythmia. Despite pvc’s at peak exercise, there were no genetic variants in genes responsible for CPVT, such as *RYR2* and *CASQ2*. Importantly, we did not observe any signs of cardiac remodeling in any of the carriers of the genetic variant, and the predominant cardiac phenotype linked to the variant was arrhythmic. However, it leaves the opportunity for the later development of dilated cardiomyopathy, as described previously in association with this genotype. The described case identifies the *ABCC9* gene as a potential causative candidate for inherited arrhythmic syndromes and underscores the significance of the use of broad target genetic panels in patients with cardiac rhythm abnormalities and no structural heart alterations.

The role of ATP-sensitive potassium currents in normal heart physiology has been illustrated by animal studies. Thus, K_ir_6.1^−/−^ and SUR2^−/−^ mice demonstrated baseline arterial hypertension, coronary artery vasospasm, and predisposition to sudden cardiac death (Chutkow et al., 2002). In contrast, in experimental animal models, K_ATP_ GOF resulted in an increased concentration of intracellular calcium and massive cytosolic calcium overload, hypercontractility, and the development of heart failure upon stress (Levin et al., 2016). These data are well in line with the stress-induced nature of the arrhythmogenic episodes in our patient and require deeper molecular studies on *ABCC9* LOF variants to identify the proper anti-arrhythmogenic strategies. There is also a possibility that arterial hypertension, observed in a patient, contributed to the arrhythmic manifestation of the genotype. Importantly, hypertension was also reported in the patient’s father carrying the same *ABCC9* variant. A similar observation can be noted for hypokalemia, observed in a patient during acute arrhythmogenic episodes. Due to abnormal K_ATP_ function, patients with *ABCC9* mutations are often reported to have a low potassium serum level which, by itself, can be a severe provocative factor for triggering ventricular fibrillation (Feest and Wrong, 1991).

Our study has several limitations. First, the number of genes tested was limited to 172 which potentially leave the possibility that other genetic causes are not well known or yet described in connection to arrhythmic disorders and cardiomyopathies can be linked to the observed phenotype. The increased number of genes studied, or the use of an exome sequencing approach potentially, will allow in excluding another genetic background linked to the patient’s phenotype. Another important limitation is the lack of functional studies for the variant described and the inability to make a strong conclusion regarding the impact of the variant on the phenotype in the genotype-positive family members. Of note, this variant was previously characterized as LOF due to the decrease in the channel expression (Bienengraeber et al., 2004). Finally, the complex effect of *ABCC9* genetic variants should be properly estimated in animal model studies, such as that conducted previously (Chutkow et al., 2002; Miki et al., 2002).

To conclude, we presented the second case of the heterozygous *ABCC9* LOF variant in a patient with arrhythmogenic cardiac phenotype, arterial hypertension, and no signs of structural heart diseases. This case expands the spectrum of *ABCC9*-related disorders and improves our understanding of the clinical consequences of K_ATP_ dysfunction.

## Data Availability

The datasets for this article are not publicly available due to concerns regarding participant/patient anonymity. Requests to access the datasets should be directed to the corresponding author.
