# Supplementary material for: Case Report: Loss-of-Function ABCC9 Genetic Variant Associated With Ventricular Fibrillation
Source: Front Genet. 2022 Apr 13;13:718853. doi: 10.3389/fgene.2022.718853 (PMC9044080; doi:10.3389/fgene.2022.718853)
Supplement: Supplementary file 1 [file Table1.DOCX]

| ABCC9 | FHL1 | LRRC10 | RBM20 |
| --- | --- | --- | --- |
| ACADVL | FHL2 | LZTR1 | RIT1 |
| ACTA1 | FHOD3 | MAP2K1 | RRAS |
| ACTC1 | FKRP | MAP2K2 | RYR2 |
| ACTN2 | FKTN | MIB1 | SALL4 |
| ACVR2B | FLNA | MMP21 | SCN10A |
| AGK | FLNC | MRAS | SCN1B |
| AKAP9 | FXN | MYBPC3 | SCN2B |
| ALPK3 | GAA | MYBPHL | SCN3B |
| ANK2 | GATA4 | MYH6 | SCN4B |
| ANKRD1 | GATA5 | MYH7 | SCN5A |
| ANO5 | GATA6 | MYL2 | SCNN1G |
| BAG3 | GATAD1 | MYL3 | SDHA |
| BRAF | GDF1 | MYL4 | SGCD |
| CACNA1C | GJA5 | MYLK2 | SHOC2 |
| CACNA2D1 | GLA | MYOF | SLMAP |
| CACNB2 | GPD1L | MYOM1 | SNTA1 |
| CALM1 | HAND1 | MYOT | SOS1 |
| CALM2 | HCN4 | MYOZ2 | SOS2 |
| CALM3 | HFE | MYPN | SPEG |
| CALR3 | HRAS | NEBL | SPRED1 |
| CASQ2 | ILK | NEXN | SYNE1 |
| CAV3 | ISPD | NF1 | SYNM |
| CBL | JPH2 | NKX2-5 | SYNPO2L |
| CDH2 | JUP | NKX2-6 | TAZ |
| CMYA5 | KCNA5 | NPPA | TBX20 |
| CRELD1 | KCND3 | NRAS | TBX5 |
| CRYAB | KCNE1 | NUP155 | TCAP |
| CSRP3 | KCNE2 | PDLIM3 | TECRL |
| CTNNA3 | KCNE3 | PKD1L1 | TGFB3 |
| DES | KCNE5 | PKP2 | TMEM43 |
| DMD | KCNH2 | PLEC | TMPO |
| DMPK | KCNJ2 | PLEKHM2 | TNNC1 |
| DNAAF1 | KCNJ5 | PLN | TNNI3 |
| DNAAF3 | KCNJ8 | PPA2 | TNNI3K |
| DPP6 | KCNQ1 | PPP1CB | TNNT2 |
| DSC2 | KRAS | PRDM16 | TPM1 |
| DSG2 | LAMA4 | PRKAG2 | TRDN |
| DSP | LAMP2 | PSEN1 | TRPM4 |
| DTNA | LDB3 | PSEN2 | TTN |
| DYSF | LEFTY2 | PTPN11 | TTR |
| EMD | LMNA | RAF1 | VCL |
| EYA4 | LMOD3 | RANGRF | ZIC3 |

Supplementary table 1. List of genes in targeted gene panel of 172 cardiomyopathy-associated genes.
